# Bridging the Gap Between Genetic Cardiomyopathies and Pregnancy

**DOI:** 10.1016/j.jacadv.2026.102784

**Published:** 2026-05-13

**Authors:** Karen Flores Rosario, Merna Hussien, Phoenix Grover, Courtlyn Witte, Richa Agarwal, Jenna Skowronski, Johanna Quist-Nelson, Michelle M. Kittleson, Rachel Peragallo Urrutia, Melinda B. Davis, Joanna M. Joly, Dana Spears, Julia Alvarenga Fagundes Couto, Jerome Jeffrey Federspiel, Melissa A. Daubert

**Affiliations:** a Division of Cardiology, Department of Medicine, Duke University Hospital, Durham, North Carolina, USA; b Division of Cardiology, Department of Medicine, University of North Carolina-Chapel Hill, Chapel Hill, North Carolina, USA; c Division of General Obstetrics, Gynecology & Midwifery, Department of Obstetrics and Gynecology, University of North Carolina-Chapel Hill, Chapel Hill, North Carolina, USA; d Division of Maternal Fetal Medicine, Department of Obstetrics and Gynecology, University of North Carolina-Chapel Hill, Chapel Hill, North Carolina, USA; e Division of Maternal Fetal Medicine, Department of Obstetrics and Gynecology, Duke University Hospital, Durham, North Carolina, USA; f Division of Hematology, Department of Medicine, Duke University School of Medicine, Durham, North Carolina, USA; g Department of Population Health Sciences, Duke University School of Medicine, Durham, North Carolina, USA; h Smidt Heart Institute, Cedars-Sinai Medical Center, Los Angeles, California, USA; i Division of Cardiovascular Medicine, Department of Medicine, University of Michigan, Ann Arbor, Michigan, USA; j Inova Schar Heart and Vascular, Inova Fairfax Medical Campus, Falls Church, Virginia, USA; k Division of Cardiology, Department of Medicine, Peter Munk Cardiac Centre, Toronto General Hospital, University Health Network, Toronto, Ontario, Canada; l Department of Medicine, Duke University Hospital, Durham, North Carolina, USA

**Keywords:** arrhythmogenic cardiomyopathy, genetic cardiomyopathy, peripartum cardiomyopathy, pregnancy

## Abstract

Genetic testing is increasingly common for people with cardiomyopathies and arrhythmias, with cascade screening recommended for first-degree relatives of those carrying pathogenic or likely pathogenic variants. Consequently, a growing number of women of childbearing potential have been identified as being genotype-positive for cardiomyopathy and arrhythmia-associated genes. Knowledge of genotype—particularly in those with strong family history—may impact reproductive planning and guide interventions to reduce pregnancy-related risks. Herein, we outline the current landscape of genetic cardiomyopathies and discuss pregnancy-related considerations for affected women. We examine the limitations of existing pregnancy risk stratification models and highlight the role of multidisciplinary Pregnancy Heart Teams in optimizing maternal and fetal outcomes. We also review counseling points for genotype-positive women considering pregnancy, including preconception planning, preimplantation genetic testing, antenatal surveillance, and delivery planning. Finally, we address recommendations for postpartum management and provide illustrative cases to guide clinical care in this growing population.

## Highlights

• The rising uptake of genetic testing underscores the need to understand genetic cardiomyopathies in pregnancy.
• A genotype- and phenotype-informed assessment during pregnancy can help identify at-risk individuals and guide timely initiation of therapeutics.
• Genotype-positive/phenotype-negative individuals represent a unique group that can benefit from dedicated surveillance and heart team input.

Genetic testing is becoming increasingly available to patients with cardiovascular disease (CVD), as a result of dramatic cost reductions from modern sequencing technologies, expanded insurance coverage, and direct-to-consumer marketing. Contemporaneously, the number of genetic variants known to result in cardiomyopathies continues to increase and more patients than ever have knowledge of a variant which may contribute to future disease. Genetic testing in defined phenotypes of CVD and cascade screening in families of affected individuals can provide insight into the range of probable phenotypes and become a tool for family-based risk assessment and prevention.^1^

CVD is a leading cause of preventable maternal death in the United States, with up to 40% attributable to cardiomyopathies.^2^ Pregnancy is a hemodynamically stressful state and women with a genetic predisposition to cardiomyopathy are at higher risk of developing the phenotype as a result.^3^ Given the intersection between increasingly identified genotype-positive individuals and increasing rates of maternal morbidity and mortality due to CVD in pregnancy, directive guidance is paramount to help bridge this gap and improve maternal-fetal outcomes.

As knowledge of genetic contributions to CVD has grown, women of childbearing potential have increasingly undergone CVD-specific genetic testing, either due to a personal history of CVD or due to cascade testing in the setting of a positive family history for CVD and a known familial variant. In addition, those with prior pregnancies complicated by peripartum cardiomyopathy (PPCM) may have undergone genetic counseling and testing, based on recent recommendations to do so for all women with PPCM.^4^ Counseling the patients in the aforementioned scenarios about the potential risks of a planned or ongoing pregnancy can be challenging. There is a paucity of data to inform these discussions and genetic testing results are not integrated into existing tools like the Cardiac Disease in Pregnancy Study II (CARPREG II) scoring system or the modified World Health Organization (mWHO) classification.^5^^,^^6^ Particular challenges include how to manage variants of uncertain significance (VUS), when to retest, and how to counsel and risk stratify asymptomatic genotype-positive/phenotype-negative (GP/PN) women in the context of pregnancy. Pregnancy heart teams encompassing obstetrics, maternal-fetal medicine, cardiologists, cardiovascular genetics specialists, anesthesiologists and genetic counselors play an important role in incorporating family history and genetic insights into comprehensive preconception counseling and care planning.^7^

## Genetic cardiomyopathies: mechanisms and inheritance

Genetic cardiomyopathies are commonly inherited in an autosomal dominant pattern, in which affected individuals are heterozygous for a pathogenic variant, and heterozygosity is sufficient to cause disease. Consequently, first-degree relatives—including parents, siblings, and children—have a 50% chance of carrying the same variant. Although many affected individuals have an affected parent, pathogenic variants can also occur de novo in the proband.^8^

Other inheritance patterns, although less common, also contribute to genetic cardiomyopathies. Autosomal recessive disease occurs when individuals inherit variants only pathogenic when biallelic. Examples include recessive dilated cardiomyopathy (DCM) caused by variants in *JPH2* and *TNNI3*, and *Naxos* disease resulting from biallelic *JUP* variants, characterized by arrhythmogenic right ventricular cardiomyopathy (ARVC) with palmoplantar keratoderma and woolly hair.^9^, ^10^, ^11^ Rarely, cardiomyopathies follow X-linked inheritance, in which hemizygous pathogenic variants on the X chromosome primarily affect biological males, whereas female carriers may exhibit a milder phenotype. For example, Duchenne muscular dystrophy, caused by *DMD* variants, illustrates this pattern where female carriers have elevated risk for DCM.^12^ In addition, mitochondrial genome variants can cause cardiomyopathy, but are influenced by degree of heteroplasmy, referring to the coexistence of more than 1 variant of mitochondrial genome within a single cell or tissue.^13^

The complexity of inheritance is further shaped by variable expressivity and incomplete penetrance. Variable expressivity refers to the wide spectrum of clinical manifestations among individuals with the same pathogenic variant, ranging from asymptomatic to severe disease. Incomplete penetrance may explain why some individuals with a pathogenic variant never develop cardiomyopathy. These characteristics differ across genes: for example, *LMNA*-related cardiomyopathy has 90% to 95% penetrance by age 60, whereas *MYBPC3* variants show roughly 55% penetrance over a lifetime.^14^^,^^15^ As a result, predicting age of onset, likelihood of presenting during the peripartum period, disease progression and severity remains challenging even when a genetic risk is known.

With increasing access to genetic testing, identifying a pathogenic variant in a proband carries important implications for family management. For known pathogenic variants, the American College of Medical Genetics and Genomics recommends cascade genetic testing of first-degree relatives.^16^ Cascade testing allows detection of relatives with the familial variant who are at risk for developing cardiomyopathy; these individuals may benefit from early surveillance and preventive care. Relatives who test negative for the familial variant can generally be reassured and released from long-term screening. However, identification of presymptomatic carriers can introduce psychosocial considerations, including anxiety about prognosis, survivor’s guilt in the setting of family members with more severe disease, and implications for reproductive decision-making.

In the following sections, cardiomyopathies are classified based on their clinical phenotype for the purposes of this discussion, given the considerable phenotypic overlap of some genetic variants. A single gene variant may perturb multiple cellular pathways, resulting in heterogeneous clinical presentations. For example, pathogenic variants in sarcomeric genes have been associated with phenotypes resembling both dilated and arrhythmogenic cardiomyopathy (ACM).

## Pregnancy in genetic cardiomyopathies: clinical phenotypes and outcomes

It is recommended that each of the following subtypes of genetic cardiomyopathies have preconception counseling, pregnancy care, and postnatal care from Pregnancy Heart Teams with individualized recommendations based on severity of the patient’s specific phenotype.

### Arrhythmogenic cardiomyopathy

The term ACM recognizes the change in nomenclature from ARVC to include left ventricular (LV) involvement beyond the fibrofatty replacement of the right ventricular myocardium. Such fibrofatty replacement predisposes patients to ventricular arrhythmias, ventricular dysfunction, and sudden cardiac death. ACM diagnosis is made based on a set of criteria that includes clinical presentation and imaging/tissue characteristics.^17^ The majority of ACM is inherited in an autosomal dominant pattern, and around 50% to 60% of patients with ACM may have an identifiable pathogenic variant in a gene associated with the cardiac desmosome. Moreover, there is incomplete penetrance (with age dependent penetrance ranging from 50% to 60%) and variable expressivity of these variants.^18^, ^19^, ^20^, ^21^ The most common genetic causes of ACM are pathogenic variants in desmosomal genes including PKP2, DSP, DSG2, DSC2, and JUP, with variants in PKP2 being the most common (Table 1).^16^

**Table 1:** Genetic Cardiomyopathies and Pregnancy Considerations

Cardiomyopathy	Pathogenic Variants	Preconception Considerations	Pregnancy Implications	Clinical Management	Postpartum Care
Arrhythmogenic cardiomyopathy (ACM)	Desmosomal genes: PKP2, DSP, DSG2, DSC2, JUP	Baseline echo or cMRI, ECG. ICD considerations; Medication adjustments, VT management, PGT	Higher arrhythmia risk; telemetry monitoring during delivery	Ambulatory ECG monitoring; continue beta blockers; ASA if high risk for preeclamsia	Follow-up echo, NT-proBNP, ECG, ambulatory ECG monitoring
Hypertrophic cardiomyopathy (HCM)	Sarcomeric genes: MYBPC3, MYH7Í, TNNI3, TNNT2, TPM1, MYL2, MYL3, ACTC1	Baseline echo or cMRI, ECG. Assess obstruction; Myectomy consideration; Medication adjustments; PGT	Assisted second stage; anesthesia and pressor considerations	Continue beta blockers; avoid hypovolemia and Valsalva; ASA if high risk for preeclamsia	Follow-up echo, NT-proBNP, ECG
Dilated cardiomyopathy (DCM)	TTN, LMNA, BAG3, DES, FLNC, MYH7, PLN, RBM20, SCN5A, TNNC1, TNNT2, DSP	Baseline echo or cMRI; ECG; NT-proBNP, PGT	Telemetry monitoring during delivery	Stop teratogens; control volume/afterload; rhythm control; LMWH; aspirin	Follow up echo, NTproBNP; continue HF therapy; resume GDMT per lactation plan.
Peripartum cardiomyopathy (PPCM)	TTNtvs, FLNC, DSP, BAG3 etc	Baseline echo, ECG, and NT-proBNP in future pregnancies; med adjustment; PGT if variant identified	Deliver at quaternary center	Genetic counseling and testing Pregnancy monitoring guided by mWHO	Follow-up echo, NT-proBNP; counsel on future pregnancy risk
Genotype positive, phenotype negative (GP/PN)	Any of the above	Baseline echo, ECG, ambulatory ECG monitor; consider cMRI/exercise test in select high risk groups	Delivery plan per family/genetic risk and symptoms identified during evaluation; monitor for development of phenotype	Monitor for HF, arrhythmias, or phenotype progression	Consider cardiac cMRI if not done or in the presence of new symptoms; genetic counseling for offspring

ASA = aspirin; cMRI = cardiac magnetic resonance imaging; ICD = implantable cardioverter-defibrillators; ECG = electrocardiogram; GDMT = guideline directed medical therapy; HF = heart failure; LMWH = low molecular weight heparin; mWHO = modified World Health Organization; NT-proBNP = N terminal pro-brain natriuretic peptide; PGT= preimplantation genetic testing; VT = ventricular tachycardia.

The physiologic stressors of pregnancy, including increase in adrenergic tone and hemodynamic load, may increase arrhythmia risk. Hence, pregnant women with ACM are thought to be at a higher risk of arrhythmia, ventricular dysfunction, and potential for poor pregnancy tolerance compared to pregnant women without ACM. ACM is generally classified as mWHO class II–III, with CARPREG 0 to 1 scores varying based on the presence of ventricular dysfunction and prior arrhythmias; notably, neither tool incorporates gene-specific risk.^6^^,^^22^ Prior studies in the ACM population identified the third trimester of pregnancy and the puerperium as being the highest risk periods for ventricular arrhythmias.^23^ Contemporary data from the NORDIC ARVC Register in 199 females with definite ARVC has shown that pregnancy was uneventful for the majority of women with ACM, and that the number of prior pregnancies was not associated with increased risk of ventricular arrhythmia. However, these pregnancies remained at an increased risk for complications compared with those without ACM. Women who gave birth with previously-diagnosed ACM had elevated risk of ventricular arrhythmias postpartum (HR: 13.74; 95% CI: 2.9-63; *P* = 0.001).^24^ Similarly, data from a combined Johns Hopkins/Dutch ARVC/D Registry, showed that pregnancy was generally tolerated, but 13% of pregnancies were complicated by ventricular arrhythmia and 5% by heart failure.^25^

A recent Heart Rhythm Society consensus statement highlighted recommendations on management of arrhythmias for women with ACM, proposing a Class 1 recommendation (strong recommendation) for implantable cardioverter-defibrillator (ICD) for secondary prevention in those with a history of ventricular arrhythmias and/or sudden cardiac death. In these high-risk patients, ICD implantation before conception is preferred, as device implantation during pregnancy carries additional procedural and fetal risk. For primary prevention, the Heart Rhythm Society endorses ICD implantation in accordance with existing guidelines—specifically, for patients with nonischemic cardiomyopathy and LV ejection fraction (LVEF) of ≤35%, and NYHA functional class II–III symptoms despite optimal medical therapy.^26^, ^27^, ^28^ In addition, recommendations on pharmacotherapy for management of chronic/recurrent ventricular arrhythmias include beta-blockers as the first-line agents and in those with refractory symptoms; for those with contraindications to beta-blockers, flecainide, sotalol, and mexiletine are considered (Central Illustration).^27^^,^^29^^,^^30^ Amiodarone use is considered the last resort only for life-threatening cases where no safer option exists.^27^^,^^30^ Catheter ablation can be considered during pregnancy if symptoms or hemodynamically unstable arrhythmias persist despite pharmacotherapy and was given a 2a (moderate) recommendation.^27^ A recent consensus document on pregnancy in patients with inherited myocardial disorders state that most current treatments for ACM (beta-blockers, antiarrhythmic drugs, and ICD therapies) should not be suspended due to pregnancy and that careful evaluation of rhythm and hemodynamics should be scheduled ideally preconception. For pregnant individuals with ACM, recommended monitoring follows recommendations for all cardiomyopathies during pregnancy and entails every 3 months electrocardiograms (ECGs), 24-hour ambulatory ECG monitoring, and N-terminal pro-brain natriuretic peptide (BNP) (NT-proBNP). In patients with reduced LVEF, class III/IV heart failure or a change in symptoms, more frequent monitoring, possibly monthly, with 24-hour ambulatory ECG monitoring may be considered.^7^

### Hypertrophic cardiomyopathy

Hypertrophic cardiomyopathy (HCM) is a disorder characterized by hypertrophy of any portion of the LV wall that is not explained by abnormal myocardial loading conditions or myocardial infarction. Subaortic muscular obstruction to LV outflow that can be provoked or exacerbated by exercise is present in roughly 70% of patients with HCM.^31^ HCM is the most prevalent monogenetic cardiac disorder and presents unique management challenges in pregnancy. From a genetic standpoint, HCM is most commonly ascribed to pathogenic variants in the sarcomeric genes MYBPC3 and MYH7, which together account for the majority of cases. Other sarcomeric genes—including TNNI3, TNNT2, TPM1, MYL2, MYL3, and ACTC1—contribute to a smaller subset of cases, typically comprising 1% to 5% of the genetic burden.^32^

The pathophysiologic underpinnings of HCM are now better understood and novel therapies targeting sarcomeric HCM that can decrease cardiac contractility are now common in clinical care, which makes pregnancy-specific considerations increasingly relevant as more women with HCM may be healthy enough to conceive. Hemodynamic adaptations of pregnancy—particularly increased blood volume and stroke volume—can lead to mild LV dilation and lower the dynamic outflow tract gradient, despite the decrease in systemic vascular resistance. However, reduced preload, tachycardia, or excessive vasodilation can exacerbate outflow obstruction and should be avoided.^33^

Pregnancy is generally well tolerated in HCM with overall low maternal mortality of 0.2% and no differences in outcomes between nonobstructive and obstructive HCM.^34^ HCM carries mWHO class II–III risk; although CARPREG II identifies LV outflow tract obstruction (LVOTO) and ventricular dysfunction as adverse predictors, it does not incorporate HCM-specific risk variables validated in dedicated pregnancy cohorts.^6^^,^^22^ A 2025 propensity-matched analysis demonstrated no significant difference in 10-year all-cause mortality between parous and nulliparous women with HCM.^35^ Earlier cohort data also support this. In a study of 127 women with 271 pregnancies, fewer than 10% experienced symptom exacerbation.^36^ However, rare complications can occur, particularly in those with severe hypertrophy, high LV gradient (those with a gradient of >100 mm Hg), or a personal or family history of severe arrhythmias, underscoring the need for individualized risk assessment, multidisciplinary care, and planned delivery with fetal surveillance.

In patients with LVOTO, current consensus advises myectomy before pregnancy if indicated, maintenance of euvolemia, and avoidance of cardiac myosin inhibitors due to potential teratogenicity.^7^ If these procedures are not performed before pregnancy or if LVOTO develops during pregnancy, the use of beta-blockers or calcium channel blockers in pregnant women with normal LVEF should be considered. Intrapartum hemodynamic management in HCM focuses on prompt treatment of hypotension with vasopressors, preferably phenylephrine, and volume resuscitation to maintain preload; in women with severe LVOTO, operative vaginal delivery should be considered to minimize the hemodynamic consequences of prolonged Valsalva.^33^^,^^37^ For arrhythmia management, treatment with beta-blockers is considered first-line and medications like amiodarone are to be used as a single dose in life-threatening emergencies (Central Illustration).^38^

**Central Illustration:**
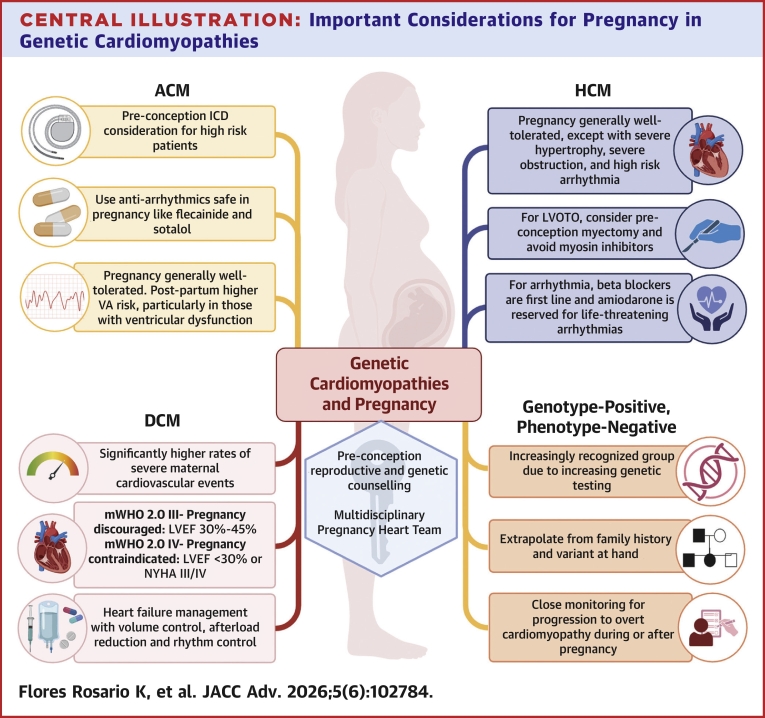
**Important Considerations for Pregnancy in Genetic Cardiomyopathies** ACM = arrhythmogenic cardiomyopathy; DCM = dilated cardiomyopathy; HCM = hypertrophic cardiomyopathy; ICD = implantable cardioverter-defibrillator; LVEF = left ventricular ejection fraction; LVOTO = left ventricular outflow tract obstruction; mWHO = modified World Health Organization; VA = ventricular arrhythmias.

### Dilated cardiomyopathy

The term DCM is defined as the presence of LV or biventricular dilatation or systolic dysfunction in the absence of abnormal loading conditions or significant coronary artery disease sufficient to cause ventricular remodeling.^39^ Key genetic contributors to DCM include truncating variants in TTN, which represent approximately 15% to 25% of cases, and pathogenic variants in LMNA, accounting for roughly 4% to 7%. Beyond these genes, a number of others have definitive or strong evidence for causality in DCM, including BAG3, DES, FLNC, MYH7, PLN, RBM20, SCN5A, TNNC1, TNNT2, and DSP (Table 1).^40^ The 2023 ESC Guidelines introduced nondilated LV cardiomyopathy as a distinct entity encompassing nonischemic LV scarring, fatty replacement, or isolated global LV hypokinesia without scarring.^41^ In the absence of nondilated LV cardiomyopathy–specific pregnancy data, we extrapolate pregnancy-related considerations from DCM pending dedicated outcomes research.^40^

Pregnant women with DCM are at an increased risk of heart failure given the hemodynamic changes in plasma volume during pregnancy. Women with DCM require adjustments in guideline-directed medical therapy (GDMT) during pregnancy due to teratogenicity, which can further increase the risk of decompensation. Women with DCM experience significantly higher rates of severe maternal cardiovascular events (heart failure, arrhythmias, and death) compared to healthy controls and those with other cardiac diseases.^42^^,^^43^ Data from large North American registries showed major adverse cardiovascular events occurred in 39% of pregnancies in women with non-PPCM, with maternal mortality rates up to 7% in high-risk groups.^44^ In these registries, pre-pregnancy LVEF <50% was the strongest predictor of poor outcome and was associated with higher rates of heart failure, further LVEF deterioration, and maternal death.^26^

The current mWHO designates women with DCM with moderate LV dysfunction (LVEF 30% to 45%) to the high-risk category (class III). In these mWHO class III scenarios, pregnancy is discouraged and not fully recommended, requiring intensive specialist monitoring by a Pregnancy Heart Team.^3^^,^^30^ Those with severely reduced ventricular function with LVEF <30% or NYHA functional class III/IV symptoms are considered mWHO class IV; meaning women in this classification have an unacceptably high risk of maternal mortality and severe morbidity, and pregnancy is highly risky and not recommended. If pregnancy is continued despite recommendations, Pregnancy Heart Team assessment at a tertiary care center is recommended with at a least monthly follow-up.^3^

Follow-up of pregnant women with DCM is influenced by severity of cardiomyopathy, symptoms, LVEF, and need for medication adjustments.^26^ However, the 2025 European guidelines recommend exams every 3 months in low-risk patients with NT-proBNP, ECG, echocardiogram and 24 h Holter monitor.^30^ Clinical reassessment is recommended bimonthly in women with mWHO II and monthly for those with mWHO III.^7^ Treatment of DCM and clinical heart failure during pregnancy is focused on controlling volume status (diuretics), afterload reduction (eg, nitrates, hydralazine), rhythm control (eg, β-blockers, digoxin), and anticoagulation if necessary (Central Illustration).^37^

### Peripartum cardiomyopathy

PPCM is defined as heart failure with systolic dysfunction (LVEF<45%) that develops toward the end of pregnancy or in the months following delivery, in the absence of known pre-existing cardiac dysfunction.^4^^,^^45^^,^^46^ PPCM incidence is higher in women of African American descent than in women of European descent and strikingly higher in populations from Haiti and Nigeria when compared with the remainder of the world.^46^^,^^47^ Approximately 15% of women with PPCM have a shared genetic predisposition with DCM and are found to have truncating loss-of-function variants in DCM genes such as TTN, FLNC, DSP, MYH6, MYH7, and BAG3.^45^ An international study of 469 women with PPCM and genetic sequencing found that over 10% of women with PPCM harbored TTNtvs, which was associated with a lower EF than other variants, but there was not a significant difference in the timing of PPCM presentation, concomitant preeclampsia, or rate of clinical recovery among the different truncating variants.^48^ Despite the growing awareness regarding the genetic underpinnings of PPCM, a recent study showed only 11% of women with PPCM was referred for a genetic evaluation.^49^ Genetic counseling and testing are now recommended for all women with PPCM (Table 1).^4^^,^^30^^,^^46^^,^^50^^,^^51^

The 2025 European Guidelines recommend discouraging subsequent pregnancy in women with PPCM and persistent LV dysfunction (EF <50%), but even among women with recovered LV function (EF ≥50%) there remains a risk for recurrent LV dysfunction, life-threatening arrhythmias and, rarely, maternal death.^30^ A baseline echocardiogram, natriuretic peptide level, and clinical assessment as well as adjustment of medical therapy are recommended before subsequent pregnancies. This information can be used to inform conversations regarding risks of PPCM relapse in future pregnancies. Recent prospective data from a European cohort suggest women with PPCM and LVEF between 40% and 50% may proceed with subsequent pregnancy under careful multidisciplinary monitoring.^50^ However, there are marked disparities in the outcomes of PPCM and subsequent pregnancies observed across different regions. In a U.S.-based retrospective study of women with PPCM and subsequent pregnancies, majority of African American descent (80%), those with nonrecovered LVEF experienced significantly higher rates of adverse outcomes (53% vs 20%) and 5-year all-cause mortality (13.3% vs 3.3%) compared with those whose LVEF recovered.^52^ Hence, close monitoring during pregnancy is recommended with repeat echocardiographic assessment of LV function and natriuretic peptide levels at end of 1st, 2nd trimester, 1 month before delivery, after delivery before discharge, 1 month postpartum, and any time symptoms develop thereafter (Table 1).^52^^,^^53^

### Genotype positive, phenotype negative

Increasingly, asymptomatic women who are contemplating pregnancy are identified as GP/PN for variants associated with CVD, meaning that they have been identified as having a potential deleterious variant through genetic testing, but do not presently have signs or symptoms consistent with CVD. Whether the hemodynamic changes of pregnancy will unmask cardiac dysfunction in these at-risk women is oftentimes unknown. As a result, these patients may have anxiety or lived experiences of family members with advanced heart failure that impacts their pregnancy experience, and therefore could benefit from a comprehensive pregnancy heart team assessment and careful shared decision-making.^7^

Most guidance on screening and surveillance for GP/PN individuals is inferred from the specific genotype variant, extrapolated from the family history, and from a baseline clinical evaluation. What is known about the specific family variant as well as the phenotype assessed on initial cardiac evaluation of these individuals can help guide future pregnancy care, cardiac monitoring plan, inform whether to avoid high intensity exercise during pregnancy due to variant-specific arrhythmogenic risk, and shape postpartum follow-up. A recent study looking at pregnancy in women with rare DCM variants showed that most asymptomatic women with rare DCM variants had uneventful pregnancies, but 10% of cases had clinically significant cardiac complications when cardiac dysfunction developed during pregnancy or shortly after, approximating the rate seen in women with cardiac disease predating pregnancy.^54^ In addition, family screening of PKP2 variant carriers has shown that 34% of GP/PN relatives progress to definite ARVC over time, suggesting that these individuals may have a dynamic prephenotypic state that can be vulnerable to phenotypic unmasking by physiological stressors, including pregnancy.^54^^,^^55^

If known before pregnancy, GP/PN patients present the ideal opportunity to implement preconception counseling and to incorporate multidisciplinary management and shared decision-making. Based on the limited information available to date, pregnancy is generally well tolerated in most GP/PN women, but the risk of developing heart failure, arrhythmias, or progression to overt cardiomyopathy due to the hemodynamic stressors of pregnancy occurs in a minority. The risk associated with these conditions is modulated by the specific genetic variant involved, the penetrance and expressivity of each variant, and the rate of development of a clinical phenotype, which complicates the development of a uniform surveillance strategy. Thus, preconception or first trimester echocardiogram, ECG, and 24-hour Holter monitor can inform risk stratification and guide the frequency of future monitoring and management in GP/PN women. In selected cases, cardiac magnetic resonant imaging (cMRI) and exercise testing can help judge contractile reserve and hemodynamic stability and further risk stratify GP/PN women before pregnancy^77^. In asymptomatic GP/PN, genotype can inform anticipatory surveillance to identify changes in NYHA functional class, arrhythmia burden, and systolic function. In GP/PN, near-term clinical outcomes are driven primarily by phenotype.

Finally, women who are found to carry a VUS in a cardiac gene should be evaluated with a careful family and clinical history, comprehensive cardiac evaluation including ECG, Holter monitor, echocardiogram, and, in selected cases, exercise stress testing and cMRI. The goal of such assessment is to determine phenotype-driven pregnancy risk, inform follow-up frequency, and plan for safe delivery (Table 1 and Central Illustration).

## Reproductive genetics and prenatal considerations

At-risk relatives benefit from a preconception genetics evaluation. Individuals with a high-risk family history of cardiomyopathy include those with a family history of a known cardiomyopathy, advanced cardiac therapies (arrhythmia ablation, heart surgery, heart transplant, and/or implantable cardiac defibrillators), sudden death before age 40, and family members with positive cardiomyopathy genetic testing. Once a pathogenic or likely pathogenic variant has been identified in a family, results can be used in a reproductive genetics evaluation.

If a genetic diagnosis is known before conception, prenatal genetic testing for monogenic disorders (PGT-M) with in vitro fertilization (IVF) can be considered. The goal of PGT-M is to identify embryos with the familial genetic change and ensure that embryos with those changes are not transferred.^56^ There are several considerations for PGT-M. A diagnostic linkage analysis protocol must be developed for PGT-M, which requires multiple genotype positive family members, and may not be available in de novo cases. PGT-M cannot be used for a VUS due to uncertainty of risk to the embryo. Presently, not all variant types are detected by PGT-M and certain variants (eg, small deletions, duplications, or complex chromosomal rearrangements) cannot be detected using current analytical strategies.^57^^,^^58^

Counseling and informed consent are essential for PGT-M. Although PGT-M decreases the likelihood of passing on genetic differences to future generations, the process of IVF paired with PGT-M can be costly and emotionally stressful and it is not without risk. There is a risk of embryo loss from the sampling procedure, and both false positive and false negative findings from embryo mosaicism (ie, the cells sampled are not representative of the entirety of the embryo).^56^ Lastly, undergoing IVF may increase the risk of morbidity and mortality in pregnancy relative to a spontaneously conceived pregnancy.^58^

For patients who elect spontaneous conception or who present during pregnancy, prenatal diagnosis is also available and may guide discussions regarding management decisions. Fetal cells can be obtained during pregnancy via chorionic villus sampling (CVS) or amniocentesis for diagnostic testing. The procedural loss rates are generally higher for CVS than amniocentesis (on the order of 1/200 vs 1/1,000), with at least part of this higher rate for CVS likely due to higher rates of spontaneous loss during the first trimester.^59^ Because the cells obtained by CVS are placental while the cells obtained by amniocentesis are fetal, there is risk of misdiagnosis from confined placental mosaicism with CVS.

## Preconception care in women with or at risk for genetic cardiomyopathies

Tailored discussions about reproductive health should ideally start early after the diagnosis of genetic cardiomyopathies, and particularly before pregnancy, in women of childbearing potential. When pregnancy is desired, pregnancy planning can lower the risk of adverse pregnancy outcomes.^60^ The goal of preconception care for women with genetic cardiomyopathies is to offer a personalized pregnancy risk stratification, assess cardiac risk stratified by age and familial predisposition, allow for multidisciplinary education and counseling on maternal, fetal, and genetic transmission outcomes, and plan to optimize such outcomes based on shared decision-making, similar to women with any CVD.^37^ Involvement of genetic counselors, cardiologists, and maternal-fetal medicine specialists facilitates the interpretation of genotype information based on family history or known pathogenic variants.

Preconception care should also include a thorough review of current medications and an opportunity to assess for overt clinical disease among GP/PN women or monitor for progression after making necessary medication changes before pregnancy in women with genetic cardiomyopathy. Such changes may include discontinuing angiotensin-converting enzyme inhibitor, angiotensin receptor blockers, mineralocorticoid receptor antagonists, sodium glucose co-transporter 2 inhibitor, ivabradine, and vericiguat and replacing them with medications known to be safer in pregnancy when possible, such as beta-blockers, hydralazine, and nitrates. When able, a baseline pre-pregnancy assessment of potential teratogenic medications allows for counseling on risks for future pregnancy.

## Pregnancy care and delivery planning

Pregnancy is a time of substantial physiological changes, including an increase in stroke volume and maternal heart rate, and decrease in vascular resistance. Fluid shifts in the peripartum and postpartum period contribute to labile peripartum blood pressure.^37^ During labor, cardiac output increases by 50% to 80% compared to prelabor values and these changes take anywhere from 48 hours to 2 weeks in resolution. In the immediate postpartum period, autotransfusion from the uteroplacental circulation and increased venous return from decompression of the inferior vena cava lead to a rise in central venous pressure and can lead to peripartum decompensation.

For patients with a genetic cardiomyopathy, Pregnancy Heart Teams play an essential role in addressing specific labor and delivery considerations for safely navigating these physiologic changes. Specific recommendations are based on the underlying pathology, but all multidisciplinary care plans should address several basic management questions.

### What can be done to optimize care during pregnancy?

Medication adjustment, serial cardiac imaging, and preeclampsia surveillance are the hallmarks of treatment of genetic cardiomyopathy during pregnancy. For patients who enter pregnancy on GDMT, adjustment of medications to avoid teratogenic medications is essential. However, medication transition can cause a reflex hypertensive event or heart failure decompensation. For patients with severe cardiomyopathy or symptomatic changes, inpatient admission for medication adjustment may be needed. Women with a pacemaker or defibrillator should be identified before delivery to ensure timely device surveillance during pregnancy and to determine degree of pacing dependence or arrhythmia burden through pregnancy to optimize delivery planning.

During pregnancy, serial imaging, typically with transthoracic echocardiography, should be undertaken. For patients with severe disease, or worsening symptoms, more frequent imaging should be performed. Low dose aspirin (in the United States, typically 81 mg or 162 mg) should be recommended starting at 12 weeks to reduce preeclampsia risk, given the higher risk of morbidity and mortality that occur when patients with cardiomyopathy have concomitant preeclampsia.^61^^,^^62^ The use of aspirin for GP/PN pregnant women remains an active area of investigation and should be guided by risk factors for preeclampsia.^30^ Although GP/PN are at a higher risk than GN/PN individuals, the benefits of aspirin in reducing preeclampsia risk in this specific population has not been rigorously evaluated and further research is needed. These recommendations reflect expert consensus and limited retrospective evidence; prospective genotype- and phenotype-informed cohort studies defining optimal imaging timing and frequency are needed.

### When and how is delivery recommended?

For most pregnant women with CVD, full-term delivery (39 weeks or beyond) is recommended; delaying delivery to this point decreases the risk of neonatal morbidity, and for most maternal cardiac disease conditions, there is no evidence earlier delivery provides maternal benefit. However, early delivery may be warranted for those with worsening cardiac function, increasing burden of arrhythmias, or development of preeclampsia. For most women, vaginal delivery is preferred unless hemodynamic instability or obstetric indications require cesarean delivery.^30^ Neuraxial anesthesia (epidural or spinal) is recommended for most patients not only for analgesia but also to limit catecholamine surges.

Patients at risk of cardiac decompensation should have clear action plans on first line management guided by pregnancy heart teams. Patients whose condition predisposes them to arrhythmias would benefit from cardiac telemetry monitoring intrapartum and in the immediate postpartum period, and intrapartum electrolyte optimization. For patients at high risk of intrapartum decompensation, invasive hemodynamic monitoring with arterial lines can be valuable. For those in which avoiding Valsalva may be appropriate; vaginal delivery can still be offered via an assisted second stage of labor. In this approach, patients labor spontaneously or via induction until they are fully dilated, then are allowed to labor down to a level in which application of instruments can be applied and the provider delivers the fetal head and body without maternal pushing.

American College of Obstetricians and Gynecologists recommends that cardiomyopathies be identified in early pregnancy and that affected individuals should give birth in the appropriate level hospital.^63^ Referring providers should evaluate whether a certain facility has the capacity to care for specific cardiac disorders (eg, Pregnancy Heart Team, obstetrical anesthesia expertise, telemetry on labor and delivery, and capacity for extracorporeal membrane oxygenation).

## Breastfeeding

Breastfeeding decision in women with genetic cardiomyopathy requires an individualized approach guided by maternal hemodynamic status, phenotypic severity, and medication profile. Most first-line heart failure medications—including beta-blockers, angiotensin-converting enzyme inhibitors, most angiotensin receptor blockers (with the exception of candesartan), and mineralocorticoid receptor antagonists—are compatible with breastfeeding based on available pharmacokinetic and safety data. Angiotensin receptor neprilysin inhibitor therapy has no documented evidence of human harm during lactation, although animal data suggest breast milk transfer, and caution is advised pending further evidence. Sodium glucose co-transporter 2 inhibitors are not recommended during breastfeeding given theoretical risks of renal toxicity in the neonate and absence of safety data.^26^^,^^30^^,^^53^ For symptomatic women with significant cardiac dysfunction, individualized risk-benefit discussions are essential to support shared decision-making around breastfeeding. In GP/PN women, no major contraindications to breastfeeding exist, although dedicated data in this population are lacking.

## Postpartum and long-term follow-up

The immediate postpartum period is the highest risk of decompensation and close monitoring for heart failure signs and symptoms is recommended. In fact, most women with PPCM present in the first month postpartum with heart failure and are at a risk of complications such as cardiogenic shock, arrhythmias, and venous thromboembolism.^26^ Although routine biomarker monitoring with BNP or NT-proBNP may offer clinical insight, current guidelines lack consensus on the optimal frequency of assessment based on cardiomyopathy subtype.^64^ Nevertheless, established BNP and NT-proBNP thresholds remain valid in the pregnant and early postpartum population, despite the dynamic hemodynamic changes of pregnancy.^65^ This period also provides a crucial opportunity to reinstitute heart failure therapy, guided by the patient’s lactation goals. Furthermore, it serves as a critical juncture for counseling on future pregnancy risks, discussing contraception, establishing individualized follow-up plans, and initiating genetic evaluation when no prior testing has been performed.

## Illustrative case examples and conclusions

**Figure 1:**
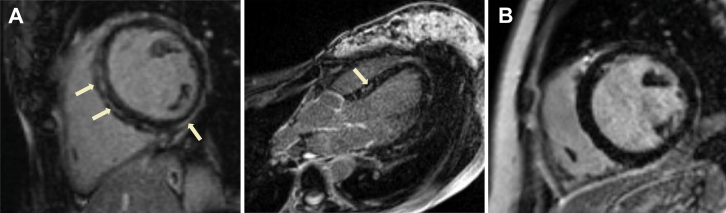
**Imaging Findings in Genetic Cardiomyopathy Cases** Cardiac magnetic resonance imaging and electrocardiogram from illustrative cases of genetic cardiomyopathies and pregnancy for (A) Case 1 and (B) Case 2. See text for details of cases.

Case 1: A 36-year-old woman with no significant personal medical history sought genetic evaluation during the third trimester of her second pregnancy after her father, who had a nonischemic cardiomyopathy and had undergone heart transplantation, was found to carry a pathogenic *FLNC* variant. Cascade screening revealed a loss-of-function *FLNC* variant (*FLNC* c.7443del [p.Lys2481Asnfs∗48]) and given concerns for 70% penetrance of FLNC variants,^66^ she established care with a Pregnancy Heart Team. Phenotypically, she had experienced no intrapartum, peripartum, or postpartum complications during her first pregnancy and remained asymptomatic throughout her second. Cardiac evaluation revealed normal NT-proBNP, normal biventricular function, and moderate (2%) premature ventricular contraction burden. She underwent induction of labor at 39 weeks and delivered a healthy infant without major complications. Telemetry intrapartum and for 24 hours postpartum did not show any arrhythmias. Postpartum cMRI revealed and LVEF of 47% and 7% late gadolinium enhancement (Figure 1). Given the increased arrhythmia risk with *FLNC* variants and the presence of symptoms, she was referred to electrophysiology for ICD consideration.

Case 2: A 33-year-old woman with a family history of sudden cardiac death developed new heart failure symptoms 5 months after a second pregnancy, which was complicated by preeclampsia. Echocardiogram revealed an LVEF of 20%, LV end diastolic diameter of 5.4 cm, and normal wall thickness. Cardiac MRI demonstrated a LVEF of 33% and a small area (1%) of subendocardial scar in the apical inferior wall due to embolic infarct. She opted not to breastfeed and was started on maximally tolerated GDMT. Family history was significant for sudden death in a maternal grandmother at age 45 and sudden death in a maternal aunt at age 30 that occurred shortly after delivery. Given her low LVEF and family history, she was fitted with a life vest for primary prevention of sudden cardiac death and referred for genetic evaluation. Genetic testing revealed a pathogenic variant in TNNT2 (c.416G>A; p. Arg139His). Her 2 young children were referred for genetic evaluation. During the most recent follow-up, her LVEF improved to 45% on GDMT. Given improvements in LVEF, ICD therapy was deferred. She was counseled against future pregnancies and underwent tubal ligation.

## Conclusions

The intersection of genetic cardiomyopathies and pregnancy represents an area of rapidly evolving clinical insight. Early involvement of a multidisciplinary Pregnancy Heart Team is essential for optimizing maternal and fetal outcomes in individuals with known or suspected genetic cardiomyopathies. This review further identifies GP/PN individuals as an emerging and under characterized subgroup, suggesting structured monitoring frameworks spanning the preconception, antepartum, and postpartum periods for this population. The cases presented herein underscore the value of integrating genetic information into pregnancy management—exemplified in Case 1 through preconception planning—and highlight the importance of incorporating genetic diagnoses into cascade screening and risk stratification strategies in affected families. To bridge the gap in pregnancy and genetic cardiomyopathies, prospective multicenter registries, genotype informed outcome studies, and particularly research in the GP/PN group are needed to translate expert-consensus into evidence and precision-based care.

## Funding support and author disclosures

The content is solely the responsibility of the authors and does not necessarily represent the official views of the National Institutes of Health. Dr Federspiel was funded by the Eunice Kennedy Shriver National Institute of Child Health and Human Development under award K12HD103083; and has consulting arrangements for Sanofi, Grifols, Noma AI, and UCB. All other authors have reported that they have no relationships relevant to the contents of this paper to disclose.
